# 2043. *In Vitro* Activity of Manogepix Against 2,810 Fungal Isolates from the SENTRY Surveillance Program (2020-2021) Stratified by Infection Type

**DOI:** 10.1093/ofid/ofac492.1665

**Published:** 2022-12-15

**Authors:** Michael D Huband, Michael Pfaller, Cecilia G Carvalhaes, Paul Bien, Mariana Castanheira

**Affiliations:** JMI Laboratories, North Liberty, Iowa; JMI Laboratories, North Liberty, Iowa; JMI Laboratories, North Liberty, Iowa; Pfizer, New York, New York; JMI Laboratories, North Liberty, Iowa

## Abstract

**Background:**

Manogepix is a new antifungal with a novel mechanism of action and potent *in vitro* activity against *Candida* (except *C. krusei*), *Aspergillus*, and rare mold isolates. The manogepix prodrug fosmanogepix is in Phase 2 development for the treatment of invasive mold infections caused by *Aspergillus* and rare molds (NCT04240886) and has completed Phase 2 studies for candidemia (NCT03604705) and *C. auris* (NCT04148287) infections. We evaluated the *in vitro* activity of manogepix and comparators against 2,810 geographically diverse fungal isolates collected in the SENTRY Surveillance Program during 2020-2021 and stratified them by infection type.

**Figure:**
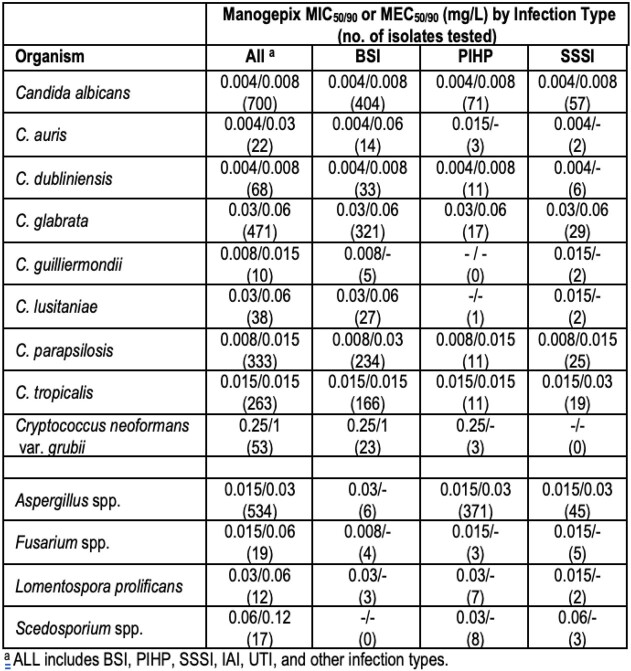

**Methods:**

Antifungal susceptibility testing of manogepix and comparator agents was conducted according to CLSI M27 (2017) and M38 (2017) guidelines. Fungal surveillance isolates were collected from medical centers located in North America (30 sites; 37.9%), Europe (30 sites; 38.4%), Asia-Pacific (11 sites; 14.7%), and Latin America (6 sites; 9.0%). Infection sites included bloodstream infections (BSI; 48.3%), pneumonia in hospitalized patients (PIHP; 19.9%), skin and skin structure infections (SSSI; 7.8%), urinary tract infections (UTI; 3.1%), intra-abdominal infections (IAI; 1.6%), and other infection types (19.3%).

**Results:**

Manogepix demonstrated potent *in vitro* activity against *Candida* spp. isolates (MIC_50/90_, 0.004-0.03/0.008-0.06 mg/L) regardless of infection type (Table). Similarly, manogepix was highly active against 534 *Aspergillus* spp. isolates (MEC_50/90_, 0.015/0.03 mg/L). Manogepix was active against 53 *Cryptococcus neoformans* var. *grubii* (MIC_50/90_, 0.25/1 mg/L), 19 *Fusarium* spp. (MEC_50/90_, 0.015/0.06 mg/L), 12 *Lomentospora prolificans* (MEC_50/90_, 0.03/0.06 mg/L), and 17 *Scedosporium* spp. isolates (MEC_50/90_, 0.06/0.12 mg/L).

**Conclusion:**

Manogepix showed potent *in vitro* activity against *Candida* spp., *Aspergillus* spp., *C. neoformans* var. *grubii*, and rare molds, including *Fusarium* spp., *L. prolificans*, and *Scedosporium* spp. isolates. Notable activity was also demonstrated by manogepix against *C. auris* isolates. Further clinical development of fosmanogepix in difficult-to-treat, resistant fungal infections is warranted.

**Disclosures:**

**Michael D. Huband, BS**, AbbVie: Grant/Research Support|Melinta: Grant/Research Support **Cecilia G. Carvalhaes, MD, PhD**, AbbVie: Grant/Research Support|Cidara: Grant/Research Support|Melinta: Grant/Research Support|Pfizer: Grant/Research Support **Mariana Castanheira, PhD**, AbbVie: Grant/Research Support|Cidara: Grant/Research Support|GSK: Grant/Research Support|Melinta: Grant/Research Support|Pfizer: Grant/Research Support|Shionogi: Grant/Research Support.

